# Outcomes of Symptomatic Anterior Large Vessel Occlusion by Initial Imaging Assessment Using Diffusion‐Weighted Imaging Versus Noncontrast Computed Tomography

**DOI:** 10.1161/SVIN.121.000170

**Published:** 2022-04-16

**Authors:** Naruhiko Kamogawa, Kanta Tanaka, Hiroshi Yamagami, Takeshi Yoshimoto, Kazutaka Uchida, Takeshi Morimoto, Hirotoshi Imamura, Nobuyuki Sakai, Nobuyuki Ohara, Yasushi Matsumoto, Masataka Takeuchi, Keigo Shigeta, Kazunori Toyoda, Shinichi Yoshimura

**Affiliations:** ^1^ Department of Cerebrovascular Medicine National Hospital Organization Osaka National Hospital Osaka Japan; ^2^ Division of Stroke Care Unit National Hospital Organization Osaka National Hospital Osaka Japan; ^3^ Department of Stroke Neurology National Hospital Organization Osaka National Hospital Osaka Japan; ^4^ Department of Stroke Neurology National Cerebral and Cardiovascular Center Suita Japan; ^5^ Department of Neurosurgery Hyogo College of Medicine Nishinomiya Japan; ^6^ Department of Clinical Epidemiology Hyogo College of Medicine Nishinomiya Japan; ^7^ Department of Neurosurgery Kobe City Medical Center General Hospital Kobe Japan; ^8^ Department of Neurology Kobe City Medical Center General Hospital Kobe Japan; ^9^ Department of Intravascular Neurosurgery Konan Hospital Sendai Japan; ^10^ Department of Neurosurgery Seisho Hospital Kanagawa Japan; ^11^ Department of Neurosurgery National Hospital Organization Disaster Medical Center Tokyo Japan

**Keywords:** computed tomography, magnetic resonance imaging, outcome, stroke, thrombectomy

## Abstract

**Background:**

We aimed to compare outcomes after stroke due to anterior circulation large vessel occlusion with initial imaging assessments using diffusion‐weighted imaging (DWI) or noncontrast computed tomography (NCCT).

**Methods:**

Among 2399 patients with large vessel occlusion stroke in a prospective, multicenter registry, patients with (1) prestroke modified Rankin Scale scores 0 to 1, (2) occlusion of the internal carotid artery or M1 segment of the middle cerebral artery, and (3) onset‐to‐hospital‐arrival time <6 hours were included. The primary effectiveness outcome was good functional outcome, defined as modified Rankin Scale scores 0 to 2 at 3 months. Safety outcomes included symptomatic intracranial hemorrhage. Intergroup biases were accounted for by mixed‐effects multivariable modeling and inverse probability of treatment weighting.

**Results:**

A total of 343 patients (130 women [37.9%]; median age, 74 years [interquartile range, IQR, 66–82 years]) were analyzed. DWI‐based assessment was performed in 217 patients, and NCCT‐based assessment was performed in 126 patients. The DWI group showed a lower baseline National Institutes of Health Stroke Scale score (*P*<0.01) and lower Alberta Stroke Program Early CT Score (*P*<0.01) than the NCCT group. Frequency of endovascular therapy was lower in the DWI group (71.9%) than in the NCCT group (84.1%; *P*<0.01). Median hospital‐arrival‐to‐arterial‐puncture time was 55 minutes (IQR, 40–77.5 minutes) in the DWI group and 55 minutes (IQR, 35–80 minutes) in the NCCT group (*P*=0.89), with similar rates of successful recanalization (77.6% versus 76.4%, respectively; *P*=0.88). Frequency of good functional outcome was 47.0% in the DWI group and 41.3% in the NCCT group (adjusted odds ratio, 1.32; 95% CI, 0.49–3.55). Symptomatic intracranial hemorrhage was encountered in 2.8% in the DWI group and 1.6% in the NCCT group (*P*=0.71).

**Conclusions:**

Potential difference in accuracy of ischemic damage assessment between the 2 modalities did not seem to affect outcomes of anterior circulation large vessel occlusion stroke.

**Registration:**

URL: https://www.clinicaltrials.gov; Unique identifier: NCT02419794.

**Nonstandard Abbreviations and Acronyms**

ASPECTS: Alberta Stroke Program Early CT Score
DWI: diffusion‐weighted imaging
ECASS: European Cooperative Acute Stroke Study
EVT: endovascular therapy
ICA: internal carotid artery
ICH: intracranial hemorrhage
IVT: intravenous thrombolysis
LKW: last known well
LVO: large vessel occlusion
mRS: modified Rankin Scale
NCCT: noncontrast computed tomography
NIHSS: National Institutes of Health Stroke Scale
RESCUE: Recovery by Endovascular Salvage for Cerebral Ultra‐acute Embolism
SITS‐MOST: Safe Implementation of Thrombolysis in Stroke‐Monitoring Study
THRACE: Thrombectomie des Artères Cerebrales

**Clinical Perspective**

**What Is New?**

- We compared outcomes after stroke attributed to anterior circulation large vessel occlusion between diffusion‐weighted imaging–based and noncontrast computed tomography–based ischemic lesion assessments using data from the RESCUE (Recovery by Endovascular Salvage for Cerebral Ultra‐acute Embolism)‐Japan Registry 2.
- Diffusion‐weighted imaging–based assessment did not affect the hospital‐arrival‐to‐intravenous‐thrombolysis‐initiation time, hospital‐arrival‐to‐arterial‐puncture time, or hospital‐arrival‐to‐successful‐canalization time compared with noncontrast computed tomography–based assessment.
- Frequency of modified Rankin Scale scores 0 to 2 at 3 months was 47.0% in the diffusion‐weighted imaging group and 41.3% in the noncontrast computed tomography group (adjusted odds ratio, 1.32; 95% CI, 0.49–3.55).

**What Are the Clinical Implications?**

- The potential difference in the accuracy of ischemic damage assessment between diffusion‐weighted imaging and noncontrast computed tomography may not be large enough to affect outcomes after anterior circulation large vessel occlusion stroke.

In the emergent care of patients with ischemic stroke attributed to anterior circulation large vessel occlusion (LVO), measurements of the extent of the ischemic lesion with the Alberta Stroke Program Early CT Score (ASPECTS) by noncontrast computed tomography (NCCT) or diffusion‐weighted imaging (DWI) remain the mainstays in treatment decisions for endovascular therapy (EVT).^1^ In data from the THRACE (Thrombectomie des Artères Cerebrales) randomized trial that evaluated the efficacy of mechanical thrombectomy after intravenous thrombolysis (IVT) in patients with LVO stroke, magnetic resonance imaging (MRI)–based and CT‐based selection showed similar treatment time intervals without affecting functional outcomes.^2^ Because treatment decisions for EVT are affected by innumerable attributes of patients, including age, functional status before stroke onset, or comorbidity as well as stroke severity and brain imaging findings, data from genuine clinical settings in which patient selection for EVT was made by a treating physician would be helpful when considering efficient care systems for stroke in each circumstance.

Here, we compared outcomes after stroke due to anterior circulation LVO between DWI‐based and NCCT‐based ischemic lesion assessments using data from the RESCUE (Recovery by Endovascular Salvage for Cerebral Ultra‐acute Embolism)‐Japan Registry 2.^3^, ^4^, ^5^, ^6^, ^7^, ^8^

## Methods

Data supporting the findings of this study are available from the corresponding author on reasonable request and with approval from the RESCUE‐Japan Registry 2 investigators.

### Study Population

The RESCUE‐Japan Registry 2 was a prospective, multicenter, observational study of ischemic stroke attributed to LVO. Participants were registered from 46 stroke centers in Japan between October 2014 and September 2016 and were followed up for 3 months. The study design has been published elsewhere.^3^, ^4^, ^5^, ^6^, ^7^, ^8^ Briefly, patients with acute LVO who were aged ≥20 years and were hospitalized within 24 hours after last known well (LKW) were enrolled. All study procedures were reviewed and approved by the ethics committees of the participating institutions. The need to obtain written informed consent from each patient was waived in this study because we used clinical information obtained from routine clinical practice. Review boards from all participating institutions approved the exemption in accordance with the ethical guidelines for Medical and Health Research Involving Human Subjects in Japan. The study was registered with ClinicalTrials.gov (NCT02419794) and the Japanese University Hospital Medical Information Network Clinical Trials Registry (UMIN000015273).

**Figure 1:**
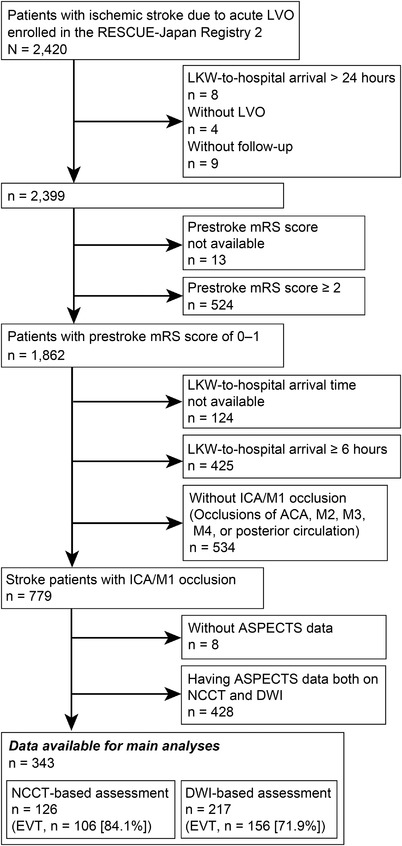
**Study flowchart**. ACA indicates anterior cerebral artery; ASPECTS, Alberta Stroke Early CT Score; DWI, diffusion‐weighted imaging; EVT, endovascular therapy; ICA, internal carotid artery; LKW, last known well; LVO, large‐vessel occlusion; mRS, modified Rankin Scale; NCCT, noncontrast computed tomography; and RESCUE, Recovery by Endovascular Salvage for Cerebral Ultra‐acute Embolism.

In the present substudy of the RESCUE‐Japan Registry 2, patients who met the following criteria were included: (1) prestroke modified Rankin Scale (mRS) score of 0 to 1, (2) LKW‐to‐hospital‐arrival time <6 hours, and (3) occlusion of the internal carotid artery (ICA) or M1 segment of the middle cerebral artery. We excluded patients who had ASPECTS data both on NCCT and DWI. Patient selection flows are shown in Figure 1.

### Treatment of LVO

Treatment modalities and indication of EVT were determined by the physician in charge. Medical management included IVT with alteplase at 0.6 mg/kg (the dose approved in Japan).^9^ EVT mainly included mechanical thrombectomy with stent retrievers or aspiration techniques; balloon angioplasty, stenting, intra‐arterial thrombolysis, or some combination thereof were performed in some cases. Any devices for the EVT procedure approved for clinical use in Japan were able to be selected at the discretion of the treating physician. For recanalization grading, the modified Thrombolysis in Cerebral Infarction scale was used, with successful recanalization defined as a modified Thrombolysis in Cerebral Infarction scale 2b or 3.^10^

### Clinical Data Collection

Baseline clinical data for the following 17 variables were collected: off‐hour hospital arrival, sex, age, atrial fibrillation, vascular risk factors (hypertension, diabetes, and dyslipidemia), past medical history (stroke before the index event or congestive heart failure), premorbid anticoagulation, premorbid antiplatelets, smoking habit, baseline systolic blood pressure, laboratory data (glucose and creatinine), LKW‐to‐hospital‐arrival time, and baseline National Institutes of Health Stroke Scale (NIHSS) score (Table 1). Off‐hour hospital arrival was defined as hospital arrival during weekends (between Friday 05:00 p.m. and Monday 08:00 a.m.), Monday to Friday between 05:00 p.m. and 08:00 a.m., and national holidays.^11^ Extent of ischemic change in the territory of the middle cerebral artery was graded according to the ASPECTS.^12^ Occlusion of the ICA, occlusion of the M1 segment of the middle cerebral artery, and tandem occlusion were determined using magnetic resonance angiography, computed tomography (CT) angiography, or digital subtraction angiography on admission. CT, MRI, and digital subtraction angiography were adjudicated locally at the treating facilities.

**Table 1:** Clinical Characteristics

	DWI based, n=217, n (%) or median (IQR)	NCCT based, n=126, n (%) or median (IQR)	*P* value	Missing data, n (%)
Off‐hour hospital arrival	141 (66.8)	80 (64.5)	0.72	8 (2.3)
Women	87 (40.1)	43 (34.1)	0.30	0 (0.0)
Age, y	74 (66–82)	75 (67–83)	0.34	0 (0.0)
Atrial fibrillation	116 (53.5)	69 (54.8)	0.82	0 (0.0)
Hypertension	121 (55.8)	73 (57.9)	0.73	0 (0.0)
Diabetes	34 (15.7)	25 (19.8)	0.37	0 (0.0)
Dyslipidemia	45 (20.7)	34 (26.9)	0.18	0 (0.0)
Stroke history before index event	12 (5.5)	8 (6.4)	0.81	0 (0.0)
Congestive heart failure	33 (15.2)	27 (21.4)	0.18	0 (0.0)
Premorbid anticoagulation	38 (17.5)	35 (27.8)	0.02	0 (0.0)
Premorbid antiplatelets	37 (17.1)	28 (22.2)	0.25	0 (0.0)
Current smoker	52 (23.9)	12 (9.5)	<0.01	0 (0.0)
Baseline systolic blood pressure, mm Hg	152 (139–166)	153 (129–172.5)	0.68	12 (3.5)
Glucose, mg/dL	125.5 (111–147)	135 (111–162)	0.11	10 (2.9)
Serum creatinine, mg/dL	0.82 (0.67–1.01)	0.86 (0.71–1.05)	0.08	2 (0.6)
LKW‐to‐hospital‐arrival time, min	95 (55–180)	77.5 (40–155)	0.01	0 (0.0)
Baseline NIHSS score	16 (12–21)	20 (16–25)	<0.01	7 (2.0)
ASPECTS	7 (5–8)	9 (7–10)	<0.01	0 (0.0)
ICA occlusion	91 (41.9)	71 (56.4)	0.01	0 (0.0)
M1 occlusion	130 (59.9)	58 (46.0)	0.01	0 (0.0)
Tandem occlusion	5 (2.3)	11 (8.7)	0.01	0 (0.0)
IVT	136 (62.7)	75 (59.5)	0.56	0 (0.0)
Hospital‐arrival‐to‐IVT‐initiation time, min	34 (19–50), n=133	31 (21–51), n=74	0.99	4 (1.1)
EVT	156 (71.9)	106 (84.1)	0.01	0 (0.0)

ASPECTS indicates Alberta Stroke Program Early CT Score; DWI, diffusion‐weighted imaging; EVT, endovascular therapy; ICA, internal carotid artery; IQR, interquartile range; IVT, intravenous thrombolysis; LKW, last known well; NCCT, noncontrast computed tomography; and NIHSS, National Institutes of Health Stroke Scale.

### Outcomes

The primary effectiveness outcome was good functional outcome, defined as mRS scores of 0 to 2 at 3 months after onset. Other effectiveness outcomes were severe disability (mRS score 5) or death at 3 months and death within 3 months.

Safety outcomes were any intracranial hemorrhage (ICH) and symptomatic ICH within 72 hours after onset. Hemorrhagic transformation was classified into 4 categories, as described by the ECASS (European Cooperative Acute Stroke Study): hemorrhagic infarction 1, hemorrhagic infarction 2, parenchymal hematoma 1, and parenchymal hematoma 2.^13^ The definition of symptomatic ICH was based on the ECASS II criteria, ECASS III criteria, and SITS‐MOST (Safe Implementation of Thrombolysis in Stroke‐Monitoring Study) criteria.^14^, ^15^, ^16^

### Statistical Analysis

Data between the patients with DWI‐based assessments and those with NCCT‐based assessments are summarized as median (interquartile range [IQR]) for continuous variables and as frequencies and percentages for categorical variables. Statistical differences between the 2 groups were assessed using the Mann‐Whitney *U* test or Fisher exact test, as appropriate. We used the Cochran‐Mantel‐Haenszel test to analyze a difference in the distribution of mRS scores. We constructed logistic regression models for effectiveness outcomes. Fixed‐effects covariates for multivariable adjustment were sex, age, LKW‐to‐hospital‐arrival time, NIHSS score, ICA occlusion, IVT, and EVT. We constructed mixed‐effects models considering center identifiers as a random effect. For each model, the odds ratio with 95% CI was calculated using the NCCT group as reference.

To reduce biases between DWI‐based and NCCT‐based assessments, we applied inverse probability of treatment weighting with the mixed‐effects logistic models. The propensity score for each group was estimated using a logistic regression model, which included all 17 baseline variables. The resultant model yielded a c‐statistic of 0.73 and a Hosmer‐Lemeshow χ^2^ statistic of 4.84 (*P*=0.77). After calculating weight values by inverse probability of treatment weighting estimators (1/propensity score for DWI‐based assessment; 1/[1–propensity score] for NCCT‐based assessment), weight was trimmed at the first and 99th percentiles to avoid extreme weight.^17^ Thereafter, data balancing was assessed using absolute standardized differences, all of which were within the margin of 0.10 (Supplemental Figure SI).^18^ Exploratory subgroup analysis for good functional outcome was performed on the basis of patient characteristics, including sex, age (<75 or ≥75 years), atrial fibrillation (yes or no), hypertension (yes or no), diabetes (yes or no), dyslipidemia (yes or no), congestive heart failure (yes or no), premorbid antithrombotic medication (yes or no), LKW‐to‐hospital‐arrival time (<90 or ≥90 minutes), baseline NIHSS score (<15 or ≥15), IVT (yes or no), and EVT (yes or no). Their interaction with the imaging modality (DWI‐based assessment or NCCT‐based assessment) was evaluated by constructing the fixed‐effects multivariable models with 2‐way interaction terms (imaging modality‐by‐subgroup). Sensitivity analyses were performed on the effectiveness outcomes for the patient subgroup treated with EVT and the subgroup that received IVT.

All reported *P* values were 2‐tailed, with values of *P*<0.05 considered statistically significant. Quantitative variables were handled as continuous in logistic models. Pairwise deletion was used for missing data handling. All analyses were performed using the Stata/IC statistical package, version 15.1 (Stata Corp LP, College Station, TX).

## Results

### Patient Characteristics

The flowchart is shown in Figure 1. The distribution of modalities used for ASPECTS measurement by registering centers is shown in Supplemental Figure SII. Among the 2399 patients in the RESCUE‐Japan Registry 2 data set, data from 343 patients with stroke admitted within 6 hours after onset and with occlusions of the ICA or M1 (130 women [37.9%], median age 74 years [IQR, 66–82 years]) were available for analyses. Off‐hour hospital arrival occurred in 221 (65.9%) patients. Median LKW‐to‐hospital‐arrival time was 90 minutes (IQR, 50–165 minutes), and the median baseline NIHSS score was 18 (IQR, 14–23). IVT was implemented in 211 patients (61.5%).

Patients for whom ASPECTS data were available based on both NCCT and DWI (n=428) were excluded in the patient selection process. Distributions of clinical characteristics of the excluded patients and analyzed patients are summarized in Supplemental Table SI. Atrial fibrillation was more frequent (53.9% versus 44.6%), the baseline NIHSS score was higher (median 18 [IQR, 14–23] versus 17 [12–22]), and IVT was more common (61.5% versus 52.3%) in the analyzed patients than in the excluded patients. EVT implementation was significantly more frequent in the analyzed data set (76.4% versus 66.4%).

Ischemic lesion assessment was based on NCCT in 126 patients (36.7%) and on DWI in 217 patients (63.3%). Clinical characteristics in the NCCT and DWI groups are shown in Table 1. Compared with the NCCT group, the DWI group less frequently received premorbid anticoagulation (*P*=0.01) and included a higher proportion of current smokers (*P*<0.01). The DWI group showed a longer LKW‐to‐hospital‐arrival time (*P*=0.01), a lower baseline NIHSS score (*P*<0.01), and lower ASPECTS (*P*<0.01) than the NCCT group. ICA occlusion (*P*=0.01) and tandem occlusion (*P*=0.01) were both more prevalent in the NCCT group. The frequency of IVT and hospital‐arrival‐to‐IVT‐initiation time did not differ between groups. The number of patients with NIHSS scores ≤5 was 12 (5.6%) in the DWI group and 0 in the NCCT group (*P*<0.01).

### Endovascular Procedures

**Figure 2:**
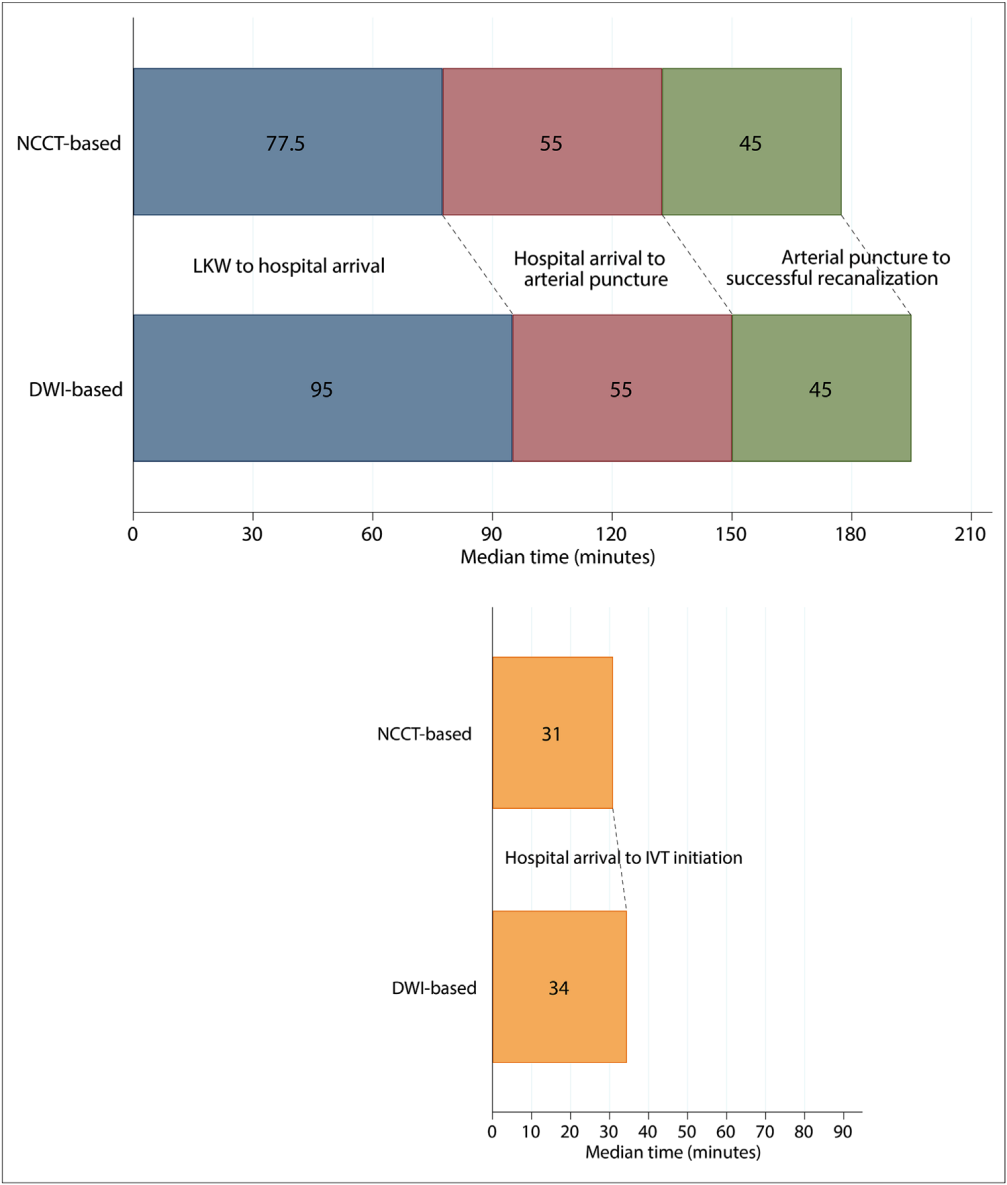
**Visual summary of median time intervals**. DWI indicates diffusion‐weighted imaging; IVT, intravenous thrombolysis; LKW, last known well; and NCCT, noncontrast computed tomography.

Among the 343 patients analyzed, EVT was performed in 262 patients (76.4%; including 226 cases with mechanical thrombectomy, 65.9%). The rate of EVT implementation was lower in the DWI group (71.9%) than in the NCCT group (84.1%, *P*=0.01; Table 1). Median hospital‐arrival‐to‐arterial‐puncture time was 55 minutes (IQR, 40–77.5 minutes) in the DWI group and 55 minutes (IQR, 35–80 minutes) in the NCCT group (*P*=0.89), with comparable rates of successful recanalization (77.6% versus 76.4%, respectively; *P*=0.88). Between the 2 groups, techniques in mechanical thrombectomy and other endovascular procedures and treatment time intervals were similar (Table 2, Figure 2).

**Table 2:** Procedural Data in Patients Who Underwent EVT

	DWI‐based EVT (n=156), n (%) or median (IQR)	NCCT‐based EVT (n=106), n (%) or median (IQR)	*P* value
Stent retriever*	88 (56.4)	66 (62.3)	0.37
Aspiration^†^	84 (53.9)	56 (52.8)	0.90
Other endovascular procedures	27 (17.3)	10 (9.4)	0.10
Balloon angioplasty	13 (8.3)	7 (6.6)	
Intracranial stenting	0 (0.0)	1 (0.9)	
Intra‐arterial thrombolysis	6 (3.9)	0 (0.0)	
Carotid artery stenting	13 (8.3)	5 (4.7)	
Successful recanalization^‡^	121 (77.6)	81 (76.4)	0.88
Hospital‐arrival‐to‐arterial‐puncture time, min	55 (40–77.5)	55 (35–80)	0.89
Arterial‐puncture‐to‐successful‐recanalization time, min	45 (30–75)	45 (30–80)	0.99
LKW‐to‐successful‐recanalization time, min	235 (160–310)	225 (150–285)	0.31

DWI indicates diffusion‐weighted imaging; EVT, endovascular therapy; IQR, interquartile range; LKW, last known well; and NCCT, noncontrast computed tomography.

* Solitaire (Medtronic, Irvine, CA) in 88 patients and Trevo (Stryker Neurovascular, Fremont, CA) in 70 patients.

^†^ All aspiration catheters were Penumbra aspiration catheters (Penumbra, Alameda, CA).

^‡^ Successful recanalization was defined as modified Thrombolysis in Cerebral Infarction scale of 2b or 3.

### Outcomes

DWI‐based assessment showed a similar distribution of mRS scores to NCCT‐based assessment (*P*=0.32) (Supplemental Figure SIII). Frequency of good functional outcome was 47.0% in the DWI group and 41.3% in the NCCT group, showing no significant difference between the groups in the mixed‐effects model (adjusted odds ratio, 1.12; 95% CI, 0.65–1.93). No significant difference was also observed after adjustment with inverse probability of treatment weighting (adjusted odds ratio, 1.32; 95% CI, 0.49–3.55). Similarly, no significant differences in risks of severe disability or death were identified between groups. Effectiveness outcomes are summarized in Table 3. In subgroup analyses for good functional outcome, no significant interactions were apparent between imaging modality and any patient characteristics (Supplemental Figure SIV).

**Table 3:** Effectiveness Outcomes

	DWI based (n=217), n (%)	NCCT based (n=126), n (%)	Crude OR (95% CI)	Mixed‐effects model (95% CI)*	Mixed‐effects IPTW model (95% CI)
mRS scores 0–2 at 3 mo	102 (47.0)	52 (41.3)	1.26 (0.81–1.97); *P*=0.30	1.12 (0.65–1.93); *P*=0.68	1.32 (0.49–3.55); *P*=0.58
Death or severe disability at 3 mo	52 (23.9)	36 (28.6)	0.79 (0.48–1.29); *P*=0.34	0.91 (0.46–1.83); *P*=0.79	0.92 (0.38–2.23); *P*=0.85
Death within 3 mo	16 (7.4)	15 (11.9)	0.59 (0.28–1.24); *P*=0.16	0.83 (0.35–1.99); *P*=0.68	1.45 (0.61–3.48); *P*=0.40

DWI indicates diffusion‐weighted imaging; IPTW, inverse probability of treatment weighting; mRS, modified Rankin Scale; NCCT, noncontrast computed tomography; and OR, odds ratio.

^*^ Fixed‐effects covariates for multivariable adjustment were sex, age, last known well‐to‐hospital‐arrival time, National Institutes of Health Stroke Scale score, internal carotid artery occlusion, intravenous thrombolysis, and endovascular therapy. Center identifiers were used as a random effect.

Safety outcomes are shown in Table 4. No intergroup differences in rates of any ICH or symptomatic ICH were identified.

**Table 4:** Safety Outcomes

	DWI based (n=217), n (%)	NCCT based (n=126), n (%)	*P* value*
Any ICH	49 (22.6)	36 (28.6)	0.24
HI1 (ECASS^†^)	5 (2.3)	6 (4.8)	0.22
HI2	16 (7.4)	15 (11.9)	0.17
PH1	7 (3.2)	8 (6.4)	0.18
PH2	6 (2.8)	3 (2.4)	1.00
Any PH	13 (6.0)	11 (8.7)	0.38
Symptomatic ICH			
ECASS II criteria^‡^	6 (2.8)	2 (1.6)	0.71
ECASS III criteria^#^	5 (2.3)	2 (1.6)	1.00
SITS‐MOST criteria^¶^	3 (1.4)	0 (0.0)	0.30

DWI indicates diffusion‐weighted imaging; ECASS, European Cooperative Acute Stroke Study; HI1, hemorrhagic infarction 1; HI2, hemorrhagic infarction 2; ICH, intracranial hemorrhage; NCCT, noncontrast computed tomography; PH, parenchymal hematoma; PH1, parenchymal hematoma 1; PH2, parenchymal hematoma 2; and SITS‐MOST, Safe Implementation of Thrombolysis in Stroke‐Monitoring Study.

* Fisher exact test.

^†^ HI1, small petechiae along the margins of the infarct; HI2, confluent petechiae within the infarcted area without space‐occupying effect; PH1, blood clots ≤30% of the infarcted area with some slight space‐occupying effect; and PH2, blood clots >30% of the infarcted area with substantial space‐occupying effect.

^‡^ Any ICH with a ≥4‐point increase in National Institutes of Health Stroke Scale score from baseline.

^#^ Any ICH as the predominant cause of a ≥ 4‐point increase in National Institutes of Health Stroke Scale score from baseline.

^¶^ PH2 combined with a ≥4‐point increase in National Institutes of Health Stroke Scale score from baseline.

### Patients Treated With EVT

We analyzed effectiveness outcomes in the subgroup of patients treated with EVT (DWI group, n=156; NCCT group, n=106). Clinical characteristics in the NCCT and DWI groups are shown in Supplemental Table SII. Patients with DWI‐based EVT showed lower baseline NIHSS scores (*P*<0.01) and lower ASPECTS (*P*<0.01) than patients with NCCT‐based EVT. Good functional outcomes were seen in 55.8% of DWI group patents and 44.3% of NCCT group patients (adjusted odds ratio, 1.56; 95% CI, 0.53–4.55). There was no significant difference in risk of severe disability or death between groups (Supplemental Table SIII).

### Patients Treated With IVT

We also analyzed effectiveness outcomes in the subgroup of patients treated with IVT (DWI group, n=136; NCCT group, n=75). Patients with DWI‐based IVT showed lower baseline NIHSS scores (*P*<0.01) and lower ASPECTS (*P*<0.01) than patients with NCCT‐based IVT. There was no significant difference in good functional outcomes, risk of severe disability, or death between the groups (Supplemental Tables SIV and SV).

## Discussion

The present substudy of the RESCUE‐Japan Registry 2 involving 343 patients with early time‐window stroke attributed to anterior circulation LVO showed that DWI‐based assessment of the ischemic lesion did not affect the hospital‐arrival‐to‐IVT‐initiation time, hospital‐arrival‐to‐arterial‐puncture time, or hospital‐arrival‐to‐successful‐recanalization time compared with NCCT‐based assessment. The choice between these 2 imaging modalities did not appear to significantly impact functional outcomes or ICH rates.

Treatment time intervals were almost the same between patients with DWI‐based or NCCT‐based assessment (Figure 2). Although image‐acquisition time was not available from the RESCUE‐Japan Registry 2 data set, data‐acquisition time is usually lower with CT than MRI.^2^, ^19^ One of the reasons for the similar time intervals between the groups may be the highest number of MRI units per capita among Organization for Economic Cooperation and Development countries when this study was conducted.^20^ It can be assumed that most of the participating centers have optimized their stroke care systems based on the assumption that MRI was the highly important modality for stroke diagnosis (Supplemental Figure SII). In addition, treatment decisions can be made while MRI acquisition is progressing and distinctive ischemic changes on DWI are easier to interpret than NCCT images with subtle early ischemic changes.^2^ Additional imaging such as CT angiography or CT perfusion after NCCT might influence the time to arterial puncture. Similarly, acquisition of magnetic resonance angiography or other imaging protocols besides DWI might influence time intervals. Hospital‐arrival‐to‐arterial‐puncture times in this study were similar to post hoc data from the General or Local Anesthesia in Intra‐Arterial therapy trial, which used MRI for EVT selection.^21^

In this study, the choice between DWI‐based ischemic lesion assessment and NCCT‐based assessment did not affect functional outcomes. Compared with the NCCT group, patients with DWI‐based assessments showed lower NIHSS scores and a lower frequency of ICA occlusion. These findings suggested that the DWI group had a higher chance of achieving good functional outcome. Moreover, in a cohort of patients with stroke treated with EVT (DEFUSE 2 [Diffusion and Perfusion Imaging Evaluation for Understanding Stroke Evolution Study 2]; enrollment from 2008 to 2011), ASPECTS from DWI offered better prediction of large ischemic core and functional outcomes than ASPECTS from NCCT.^12^ However, our study found no significant associations between modality choice and outcomes. Sensitivity analyses in the subgroup of patients who underwent EVT demonstrated the same results. During our study period of 2014 to 2016, second‐generation thrombectomy devices allowing relatively high rates of successful recanalization were in use. This might have obscured potential benefits of DWI in patient selection and could have resulted in this study being underpowered. The lower EVT rate in the DWI group would be related to the milder neurological symptoms in the DWI group compared with the NCCT group (Table 1).^2^ Lower ASPECTS in the DWI group may also have been related to the lower rate of EVT implementation in this group. LKW‐to‐hospital‐arrival time was longer in the DWI group than in the NCCT group, presumably because DWI could be compared with other sequences such as fluid‐attenuated inversion recovery for onset‐time estimation in patients with stroke with unknown time of onset.^22^ This bias between the groups might also have affected the outcomes. The possibility that DWI leads to more precise patient selection remains to be determined.^19^

This study has several limitations that merit consideration. First and most critically, a large proportion of patients were excluded. In the RESCUE‐Japan Registry 2 data set, more than half of the patients with anterior circulation LVO had ASPECTS data available from both DWI and NCCT (Figure 1). In Japan, NCCT images are commonly acquired to rule out the presence of ICH before obtaining MRI during the emergent assessment of patients with stroke (Supplemental Figure SII). Because the analyzed patients more frequently underwent EVT than the excluded patients, the data analyzed in the present study might differ in clinical features from those of the general population of LVO stroke in Japan. It is also important to note that the baseline characteristics between the NCCT and DWI groups were significantly different, which limits the comparability between the 2 groups. Second, variables related to practical differences in imaging modality selection among participating centers, including door‐to‐imaging time or admission status (direct admission or transfer to a comprehensive stroke center), went unmeasured. Third, simultaneous use of other modalities including perfusion imaging was unknown. During the study period, perfusion imaging and ischemic core volumetry were barely in use in Japan. Fourth, because DWI can more accurately depict acute ischemic lesions than NCCT, DWI may have lower ASPECTS than NCCT even with the same volume of ischemic core.^23^ DWI‐based assessment might overly exclude potentially eligible patients from EVT. Although the reasons for not performing EVT were not available from the data set of this study, low ASPECTS could be a possible reason for exclusion. Such selection bias might have affected the present analysis. Fifth, the current study differed in its results of safety outcomes. Although symptomatic ICH and death have been reported to be less frequent in the DWI‐based assessment than in the CT‐based assessment, no such difference was observed in this study.^19^, ^24^ A notable difference between our study and previous reports is that previous reports included only patients who had received EVT, whereas this study included both patients who had and had not received EVT. Also, the incidences of symptomatic ICH or mortality in both groups were lower compared with the previous studies. The current study might have been statistically underpowered because of the small sample size. Sixth, missing values were not very rare. Last, other cohorts containing ICH and other neurological emergencies, as well as ischemic stroke, may show results differing from those of the present study.^25^

## Conclusions

No differences in treatment time intervals or outcomes were identified between DWI‐based and NCCT‐based ischemic lesion assessments for anterior circulation LVO stroke. Potential differences in the accuracy of ischemic damage assessment between these 2 modalities may not be large enough to affect outcomes after anterior circulation LVO stroke.

## Sources of funding

This study was supported in part by the Japan Agency for Medical Research and Development; the Japanese Society for Neuroendovascular Therapy; the Ministry of Health, Labour and Welfare of Japan; Medtronic; Stryker; and Medicos Hirata. None of the funding sources participated in any part of the study, from study conception to article preparation.

## Disclosures

Dr Tanaka reports lecturer's fees from Medico's Hirata and Stryker. Dr Yamagami reports research grants from Bristol‐Myers Squibb; lecturer's fees from Stryker, Terumo, Medtronic, Medico's Hirata, Johnson and Johnson, Bayer, Daiichi‐Sankyo, Bristol‐Myers Squibb, Boehringer Ingelheim, Takeda, and Otsuka Pharmaceutical; and membership of advisory boards for Daiichi‐Sankyo. Dr Yoshimoto reports lecturer's fees from Takeda Pharmaceutical. Dr Uchida reports lecturer's fees from Daiichi Sankyo. Dr Morimoto reports lecturer's fees from Bristol‐Myers Squibb, Daiichi Sankyo, Japan Lifeline, Kowa, Kyocera, Novartis, and Toray; manuscript fees from Bristol‐Myers Squibb and Kowa; and advisory board membership for Sanofi. Dr Imamura reports lecturer's fees from Medtronic and Stryker. Dr Sakai reports research grants from Daiichi Sankyo, Medtronic, and Terumo and lecturer's fees from Asahi Intec and Stryker. Dr Matsumoto reports personal fees from Medtronic, Stryker, Kaneka Medics, Medicos Hirata, Cerenovus, Century Medical, Fuji Systems, Takeda pharmaceutical, Otsuka Pharmaceutical, and Terumo outside the submitted work. Dr Takeuchi reports lecturer's fees from Daiichi Sankyo. Dr Toyoda reports personal fees from Daiichi Sankyo, Bayer Yakuhin, Bristol‐Myers Squibb, and Nippon Boehringer Ingelheim outside the submitted work. Dr Yoshimura reports lecturer's fee from Medtronic, Johnson & Johnson, Terumo, Bristol‐Myers Squibb, Otsuka, Boehringer Ingelheim, Daiichi Sankyo, Bayer, Sanofi, Biomedical Solutions, Kaneka Medics, Bristol‐Myers Squibb, and Stryker. The other authors report no conflicts.

## Supporting information



Supplemental Materials
