# Supplementary material for: Outcomes of Symptomatic Anterior Large Vessel Occlusion by Initial Imaging Assessment Using Diffusion‐Weighted Imaging Versus Noncontrast Computed Tomography
Source: Stroke Vasc Interv Neurol. 2022 Apr 16;2(5):e000170. doi: 10.1161/SVIN.121.000170 (PMC12778805; doi:10.1161/SVIN.121.000170)
Supplement: Supplementary file 1 — Supplemental Materials [file SVI2-2-e000170-s001.pdf]

SUPPLEMENTAL MATERIALS

Outcomes of symptomatic anterior large vessel occlusion by initial imaging assessment using DWI versus non-contrast CT

Naruhiko Kamogawa, MD[1], Kanta Tanaka, MD, PhD[2], Hiroshi Yamagami, MD, PhD[3], Takeshi Yoshimoto, MD[4], Kazutaka Uchida, MD, PhD[5], Takeshi Morimoto, MD, PhD, MPH[6], Hirotoshi Imamura, MD, PhD[7], Nobuyuki Sakai, MD, PhD[7], Nobuyuki Ohara, MD[8], Yasushi Matsumoto, MD, PhD[9], Masataka Takeuchi, MD[10], Keigo Shigeta, MD, PhD[11], Kazunori Toyoda, MD, PhD[12], Shinichi Yoshimura, MD, PhD[5].

[1]Department of Cerebrovascular Medicine, [2]Division of Stroke Care Unit, [4]Department of Stroke Neurology, and [12]Department of Cerebrovascular Medicine, National Cerebral and Cardiovascular Center, Suita, Japan

[3]Department of Stroke Neurology, National Hospital Organization Osaka National Hospital, Osaka, Japan

Department of [5]Neurosurgery, and [6]Clinical Epidemiology, Hyogo College of Medicine, Nishinomiya, Japan

Department of [7]Neurosurgery, and 8Neurology, Kobe City Medical Center General Hospital, Kobe, Japan

[9]Department of Intravascular Neurosurgery, Konan Hospital, Sendai, Japan

[10]Department of Neurosurgery, Seisho Hospital, Kanagawa, Japan

[11]Department of Neurosurgery, National Hospital Organization Disaster Medical Center, Tokyo, Japan

Supplemental figures

Figure SI. Intergroup absolute standardized difference before and after IPTW (Page 4)

Figure SII. Distribution of modalities used for ASPECTS measurement over registering centers (Page 5)

Figure SIII. Overall distribution of mRS scores at 3 months after onset (Page 6)

Figure SIV. Subgroup analysis for good functional outcome (Page 7-8)

Supplement tables

Table SI. Clinical characteristics between patients with ASPECTS data either on NCCT or DWI and those with data both on NCCT and DWI (page 9-10)

Table SII. Clinical characteristics within patients treated with EVT (Page 11-12)

Table SIII. Effectiveness outcomes in patients treated with EVT (Page 13)

Table SIV. Clinical characteristics within patients treated with IVT (Page 14)

Table SV. Effectiveness outcomes in patients treated with IVT (Page 15)

Supplemental Figure SI. Intergroup absolute standardized difference before and after IPTW

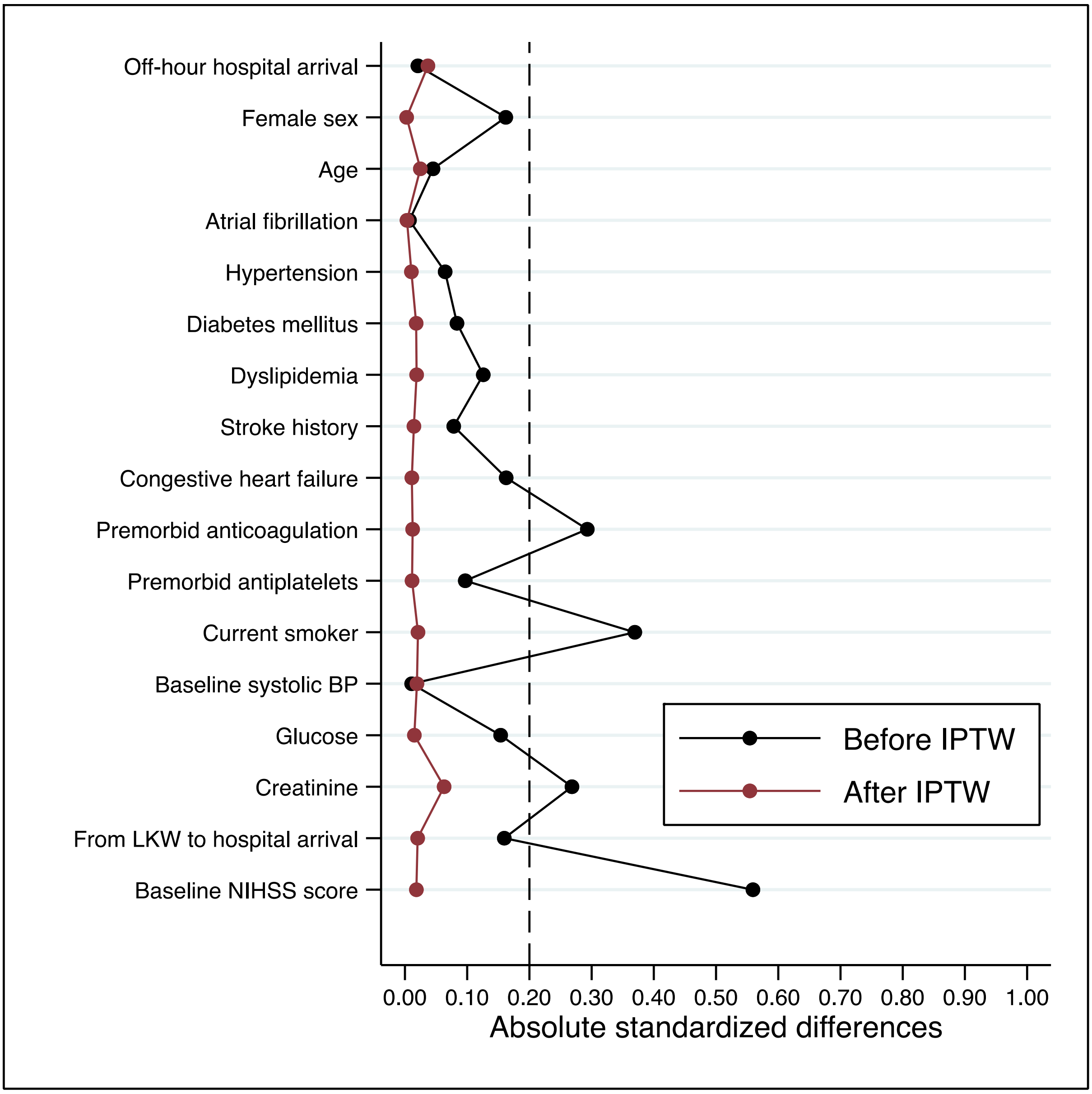

Abbreviations: BP, blood pressure; ICA, internal carotid artery; IPTW, inverse probability of treatment weighting; LKW, last known well; NIHSS, National Institutes of Health Stroke Scale.

Supplemental Figure SII. Distribution of modalities used for ASPECTS measurement among registering centers

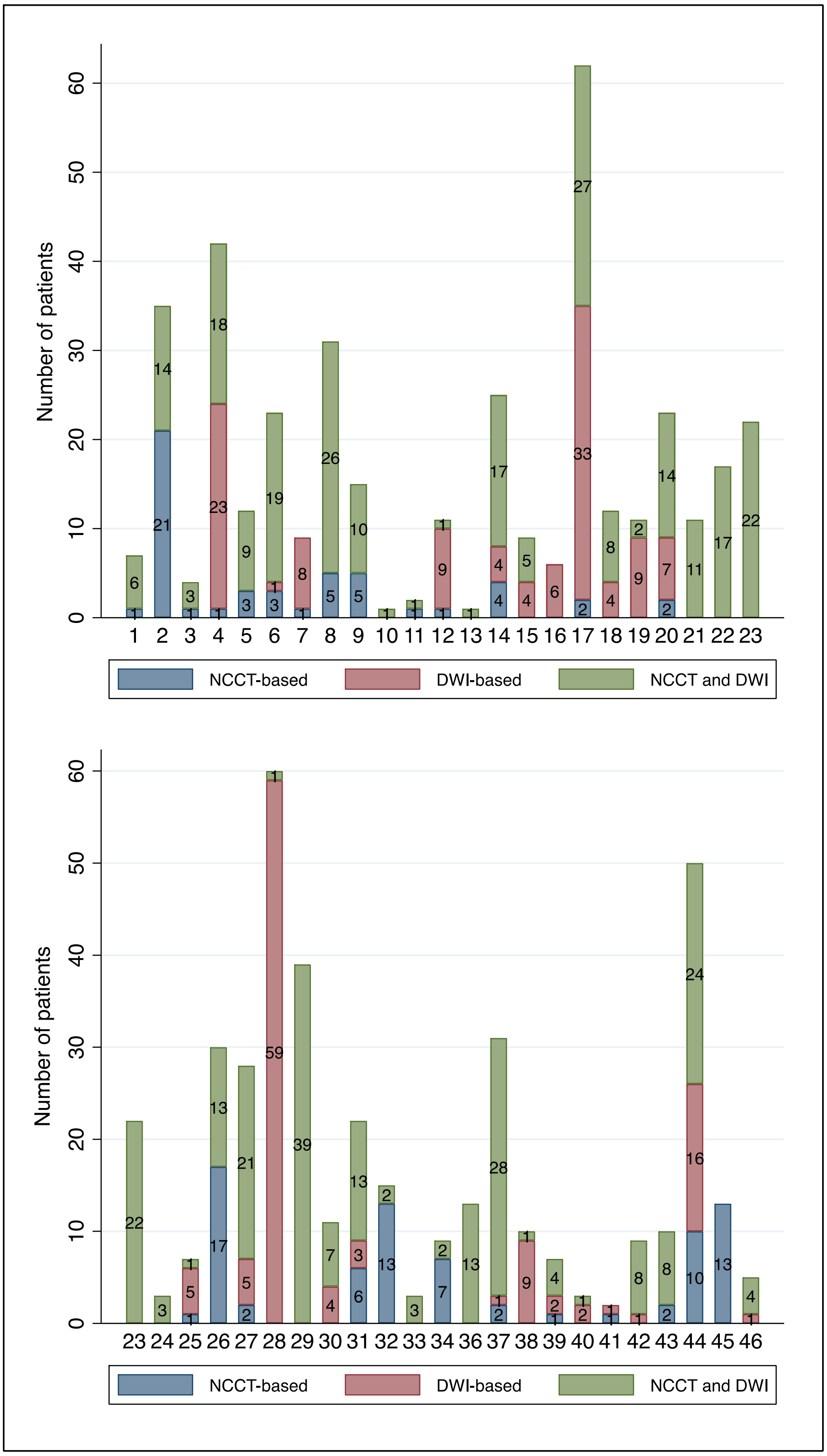

Modality distributions (n=771) over 46 centers are shown. NCCT-based: n=126; DWI-based: n=217; and both (NCCT and DWI): n=428.

Abbreviations: ASPECTS, Alberta Stroke Early CT Score; DWI, diffusion-weighted imaging; NCCT, non-contrast computed tomography.

Supplemental Figure SIII. Overall distribution of mRS scores at 3 months after onset

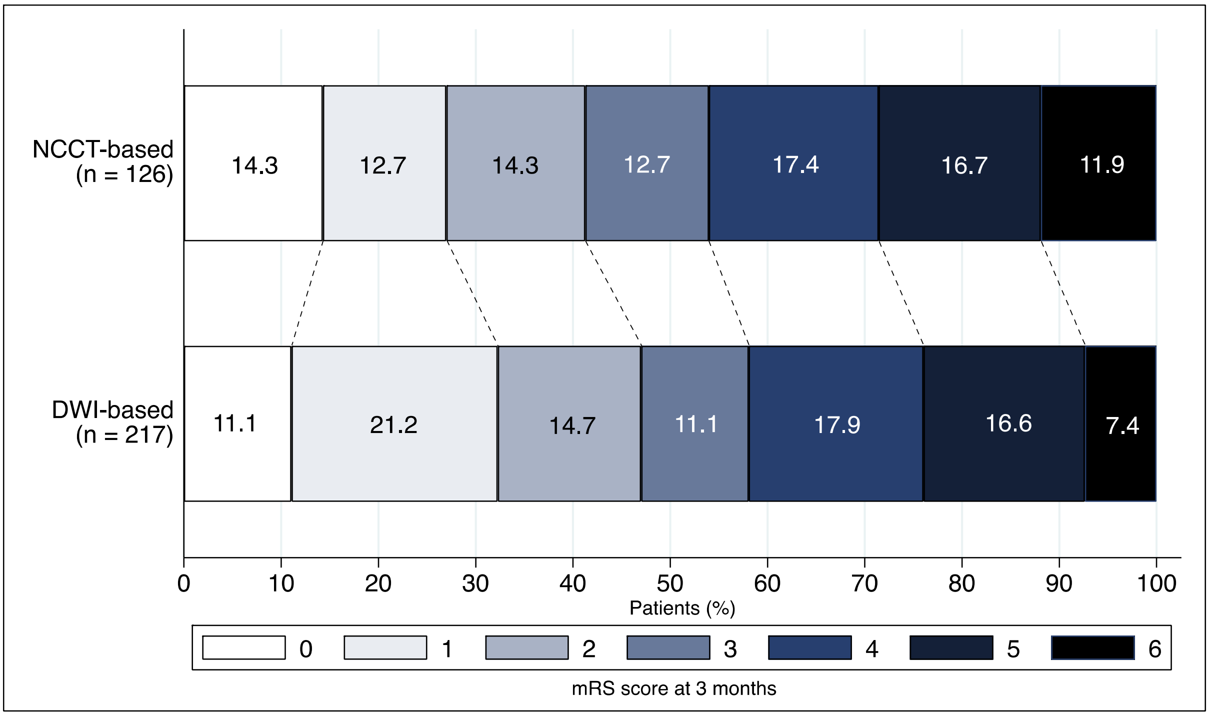

Abbreviations: DWI, diffusion-weighted imaging; mRS, modified Rankin Scale; NCCT, non-contrast computed tomography.

Supplemental Figure SIV. Subgroup analysis for good functional outcome

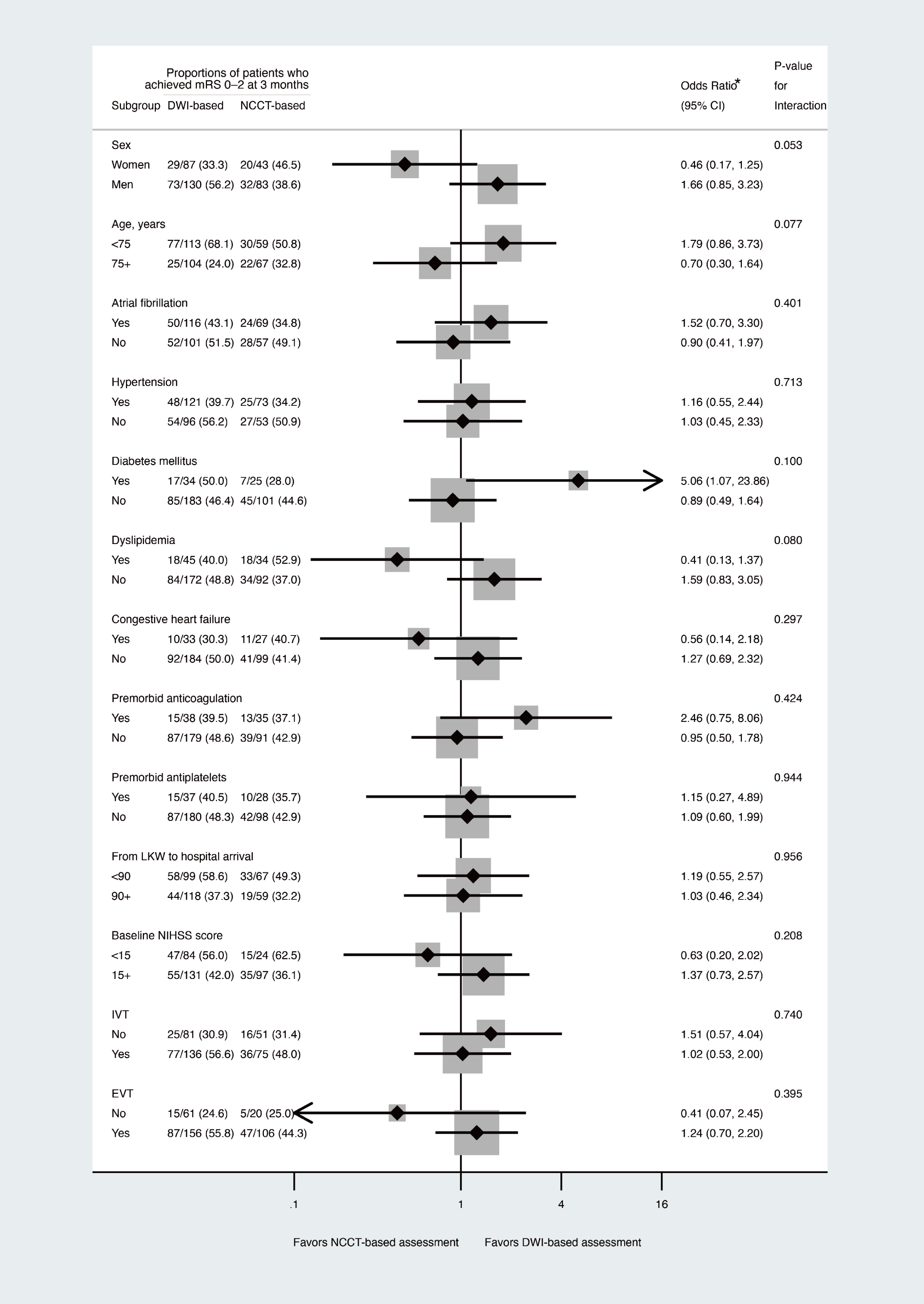

* Odds ratios were computed using fixed-effects logistic models with sex, age, LKW-to-hospital arrival time, NIHSS score, ICA occlusion, IVT, and EVT.

Abbreviations: DWI, diffusion-weighted imaging; EVT, endovascular therapy; ICA, internal carotid artery; IVT, intravenous thrombolysis; LKW, last known well; mRS, modified Rankin Scale; NCCT, non-contrast computed tomography; NIHSS, National Institutes of Health Stroke Scale.

Supplemental Table SI. Clinical characteristics between patients with ASPECTS data either on NCCT or DWI and those with data both on NCCT and DWI

	Either NCCT or DWI (n=343)	NCCT and DWI (n=428)	p-value
Off-hour hospital arrival, n (%)	221 (66.0)	273 (65.9)	1.00
Women, n (%)	130 (37.9)	154 (36.0)	0.60
Age, median (IQR), y	74.0 (66-82)	76.0 (67-82)	0.51
Atrial fibrillation, n (%)	185 (53.9)	191 (44.6)	0.01
Hypertension, n (%)	194 (56.6)	234 (54.7)	0.61
Diabetes mellitus, n (%)	59 (17.2)	80 (18.7)	0.64
Dyslipidemia, n (%)	79 (23.0)	76 (17.8)	0.07
Stroke history prior to index event, n (%)	20 (5.8)	24 (5.6)	1.00
Congestive heart failure, n (%)	60 (17.5)	59 (13.8)	0.16
Premorbid anticoagulation, n (%)	73 (21.3)	92 (21.5)	1.00
Premorbid antiplatelets, n (%)	65 (19.0)	74 (17.3)	0.57
Current smoker, n (%)	64 (18.7)	61 (14.3)	0.12
Baseline systolic blood pressure, median (IQR), mmHg	152 (137-169)	155 (136-172)	0.54
Glucose, median (IQR), mg/dl	128 (111-151)	125 (109-156)	0.73
Serum creatinine, median (IQR), mg/dl	0.8 (0.7-1.0)	0.8 (0.7-1.0)	0.56
LKW-to-hospital arrival time, median (IQR), min	90 (50-165)	95 (45-170)	0.57
Baseline NIHSS score, median (IQR)	18 (14-23)	17 (12-22)	0.04
DWI-ASPECTS, median (IQR)	7 (5-8), n=217	7 (5-9)	0.18
NCCT-ASPECTS, median (IQR)	9 (7-10), n=126	10 (7-10)	0.05
ICA occlusion (%)	162 (47.2)	196 (45.8)	0.72
M1 occlusion, n(%)	188 (54.8)	244 (57.0)	0.56
Tandem occlusion, n (%)	16 (4.7)	24 (5.6)	0.63
IVT, n(%)	211 (61.5)	224 (52.3)	0.01
Hospital arrival-to-IVT initiation time, median (IQR), min	34 (19-50)	40 (10-64)	0.13
EVT, n (%)	262 (76.4)	284 (66.4)	<0.01

Abbreviations: ASPECTS, Alberta Stroke Program Early CT Score; DWI, diffusion-weighted imaging; EVT, endovascular therapy; ICA, internal carotid artery; IQR, interquartile range;

IVT, intravenous thrombolysis; LKW, last known well; NCCT, non-contrast computed tomography; NIHSS, National Institute of Health Stroke Scale.

Supplemental Table SII. Clinical characteristics within patients treated with EVT

	**DWI-based EVT (n=156)**	**NCCT-based EVT (n=106)**	***P* value**	**Missing data, n (%)**
Off-hour hospital arrival, n (%)	106 (69.7)	66 (63.5)	0.34	6 (2.3)
Women, n (%)	50 (32.1)	35 (33.0)	0.89	0 (0.0)
Age, median (IQR), y	72 (64.5–79.5)	75 (67–83)	0.05	0 (0.0)
Atrial fibrillation, n (%)	75 (48.1)	58 (54.7)	0.31	0 (0.0)
Hypertension, n (%)	83 (53.2)	61 (57.6)	0.52	0 (0.0)
Diabetes mellitus, n (%)	27 (17.3)	22 (20.8)	0.52	0 (0.0)
Dyslipidemia, n (%)	32 (20.5)	29 (27.4)	0.23	0 (0.0)
Stroke history prior to index event, n (%)	9 (5.8)	6 (5.7)	1.00	0 (0.0)
Congestive heart failure, n (%)	24 (15.4)	24 (22.6)	0.14	0 (0.0)
Premorbid anticoagulation, n (%)	25 (16.0)	32 (30.2)	<0.01	0 (0.0)
Premorbid antiplatelets, n (%)	26 (16.7)	21 (19.8)	0.51	0 (0.0)
Current smoker, n (%)	41 (26.3)	12 (11.3)	<0.01	0 (0.0)
Baseline systolic blood pressure, median (IQR), mmHg	152 (139–166)	155 (127–172)	0.99	10 (3.9)
Glucose, median (IQR), mg/dL	128 (111–150)	133 (110–162)	0.49	10 (3.9)
Serum creatinine, median (IQR), mg/dL	0.83 (0.68–0.99)	0.87 (0.7–1.12)	0.14	2 (0.8)
From LKW to hospital arrival, median (IQR), min	90 (50–180)	70 (40–150)	0.04	0 (0.0)
Baseline NIHSS score, median (IQR)	16 (13–21)	20 (16–24)	<0.01	5 (1.9)
ASPECTS, median (IQR)	7 (6–8)	10 (7–10)	<0.01	0 (0.0)
ICA occlusion, n (%)	68 (43.6)	62 (58.5)	0.02	0 (0.0)
M1 occlusion, n (%)	92 (58.9)	47 (44.3)	0.02	0 (0.0)
Tandem occlusion, n (%)	5 (3.2)	9 (8.5)	0.09	0 (0.0)
IVT, n (%)	109 (69.9)	65 (61.3)	0.18	0 (0.0)
From hospital arrival to IVT initiation, median (IQR), min	31 (14–50), n=107	31 (17–50), n=64	0.87	3 (1.1)

Abbreviations: ASPECTS, Alberta Stroke Program Early CT Score; DWI, diffusion-weighted imaging; EVT, endovascular therapy; ICA, internal carotid artery; IQR, interquartile range;

IVT, intravenous thrombolysis; LKW, last known well; NCCT, non-contrast computed tomography; NIHSS, National Institute of Health Stroke Scale.

Supplemental Table SIII. Effectiveness outcomes in patients treated with EVT

	**DWI-based EVT (n=156)**	**NCCT-based EVT (n=106)**	**Crude OR (95% CI)**	**Mixed-effects model (95% CI)** *	**Mixed-effects IPTW model (95% CI)**
mRS score 0–2 at 3 months, n (%)	87 (55.8)	47 (44.3)	1.58 (0.96–2.60); *P*=0.07	1.25 (0.69–2.25); *P*=0.46	1.56 (0.53–4.55); *P*=0.41
Death or severe disability at 3 months, n (%)	27 (17.3)	25 (23.6)	0.68 (0.37–1.25); *P*=0.21	0.85 (0.36–2.02); *P*=0.71	1.15 (0.41–3.22); *P*=0.79
Death within 3 months, n (%)	10 (6.4)	13 (12.3)	0.49 (0.21–1.16); *P*=0.10	0.64 (0.25–1.61); *P*=0.34	1.04 (0.31–3.49); *P*=0.94

* Fixed-effects covariates for multivariable adjustment were sex, age, LKW-to-hospital arrival time, NIHSS score, ICA occlusion, and IVT. Center identifiers were used as a random effect. Abbreviations: CI, confidence interval; DWI, diffusion-weighted imaging; EVT, endovascular therapy; ICA, internal carotid artery; IPTW, inverse probability of treatment weighting; IVT, intravenous thrombolysis; LKW, last known well; mRS, modified Rankin Scale; NCCT, non-contrast computed tomography; NIHSS, National Institute of Health Stroke Scale; OR, odds ratio.

Supplemental Table SIV. Clinical characteristics within patients treated with IVT

	DWI-based IVT (n=136)	NCCT-based IVT (n=75)	p-value
Off-hour hospital arrival, n (%)	87 (64.4)	44 (59.5)	0.55
Women, n (%)	52 (38.2)	23 (30.7)	0.30
Age, median (IQR), y	72 (65-81)	75 (67-83)	0.09
Atrial fibrillation, n (%)	75 (55.1)	38 (50.7)	0.57
Hypertension, n (%)	69 (50.7)	40 (53.3)	0.77
Diabetes mellitus, n (%)	21 (15.4)	10 (13.3)	0.84
Dyslipidemia, n (%)	22 (16.2)	23 (30.7)	0.02
Stroke history prior to index event, n (%)	5 (3.7)	4 (5.3)	0.72
Congestive heart failure, n (%)	19 (14.0)	15 (20.0)	0.33
Premorbid anticoagulation, n (%)	16 (11.8)	15 (20.0)	0.15
Premorbid antiplatelets, n (%)	23 (16.9)	17 (22.7)	0.36
Current smoker, n (%)	34 (25.0)	7 (9.3)	<0.01
Baseline systolic blood pressure, median (IQR), mmHg	150 (140-163)	153 (135-173)	0.82
Glucose, median (IQR), mg/dl	127 (110-147)	132 (109-158)	0.61
Serum creatinine, median (IQR), mg/dl	0.8 (0.7-1.0)	0.9 (0.7-1.0)	0.19
LKW-to-hospital arrival time, median (IQR), min	73 (50-133)	65 (40-130)	0.12
Baseline NIHSS score, median (IQR)	16 (12-21)	20 (16-24)	<0.01
ASPECTS, median (IQR)	7 (6-8)	10 (8-10)	<0.01
ICA occlusion, n (%)	48 (35.3)	37 (49.3)	0.06
M1 occlusion, n (%)	89 (65.4)	38 (50.7)	0.04
Tandem occlusion, n (%)	2 (1.5)	4 (5.3)	0.19
Hospital arrival-to-IVT initiation time, median (IQR), min	34.5 (19.2-49.8)	30.9 (20.6-50.7)	0.78
EVT, n (%)	109 (80.1)	65 (86.7)	0.26

Abbreviations: ASPECTS, Alberta Stroke Program Early CT Score; DWI, diffusion-weighted imaging; EVT, endovascular therapy; ICA, internal carotid artery; IQR, interquartile range; IVT, intravenous thrombolysis; LKW, last known well; NCCT, non-contrast computed tomography; NIHSS, National Institute of Health Stroke Scale.

Supplemental Table SV. Effectiveness outcomes in patients treated with IVT

	**DWI-based IVT (n=136)**	**NCCT-based IVT (n=75)**	**Crude OR (95% CI)**	**Mixed-effects model (95% CI)** *	**Mixed-effects IPTW model (95% CI)**
mRS score 0–2 at 3 months, n (%)	77 (56.6)	36 (48.0)	1.41 (0.80–2.49); *P*=0.23	1.08 (0.47–2.45); *P*=0.86	1.22 (0.21–7.03); *P*=0.82
Death or severe disability at 3 months, n (%)	20 (14.7)	15 (20.0)	0.69 (0.33–1.44); *P*=0.32	0.69 (0.22–2.20); *P*=0.53	0.29 (0.03–2.54); *P*=0.26
Death within 3 months, n (%)	5 (3.7)	7 (9.3)	0.37 (0.11–1.21); *P*=0.10	0.44 (0.12–1.56); *P*=0.20	0.53 (0.02–10.68); *P*=0.67

* The fixed-effects covariates for multivariable adjustment were sex, age, time from LKW to hospital arrival, NIHSS score, ICA occlusion, and EVT. Center identifiers were used as a random effect.

Abbreviations: CI, confidence interval; DWI, diffusion-weighted MRI; IVT, intravenous thrombolysis; EVT, endovascular therapy; ICA, internal carotid artery; IPTW, inverse probability of treatment weighting; LKW, last known well; mRS, modified Rankin Scale; NCCT, non-contrast CT; NIHSS, NIH Stroke Scale; OR, odds ratio.
